# Adaptive Bidirectional Gray-Scale Center of Gravity Extraction Algorithm of Laser Stripes

**DOI:** 10.3390/s22249567

**Published:** 2022-12-07

**Authors:** Miaomiao Zhang, Zhengnan Li, Fuquan Zhang, Lidong Ma

**Affiliations:** School of Mechanical Engineering, Taiyuan University of Science and Technology, Taiyuan 030024, China

**Keywords:** line-structured light, central extraction, adaptive image processing area, upper profile, weight optimization

## Abstract

Aiming at the realization of fast and high-precision detection of the workpiece, an adaptive bidirectional gray-scale center of gravity extraction algorithm for laser stripes is proposed in this paper. The algorithm is processed in the following steps. Firstly, the initial image processing area is set according to the floating field of the camera’s light stripe, followed by setting the adaptive image processing area according to the actual position of the light stripe. Secondly, the center of light stripe is obtained by using the method of combining the upper contour with the barycenter of the bidirectional gray-scale. The obtained center of the light stripe is optimized by reducing the deviation from adjacent center points. Finally, the slope and intercept are used to complete the breakpoint. The experimental results show that the algorithm has the advantages of high speed and precision and has specific adaptability to the laser stripes of the complex environment. Compared with other conventional algorithms, it greatly improves and can be used in industrial detection.

## 1. Introduction

In the industrial production line, the real-time detection of the workpiece is an essential link in the industrial production process. However, there are some difficulties in achieving the real-time, fast and high-precision measurement of workpieces, which result from the testing environment of the factory, noise, material and testing surface. A linear-structured light measurement technology based on the triangle method is widely used in industrial measurement because it has advantages, including real-time performance, high precision and not having to touch the measuring surface. This laser scanning detection system is usually built by industrial cameras, line lasers and industrial computers. In order to realize the real-time, fast and high-precision detection of the workpiece, the pixel coordinates of the center of the light stripe are extracted by a thinning algorithm, and the pixel coordinates are converted into world coordinates by camera calibration method [1].

Usually, the methods [2] of extreme value, edge, threshold, geometric center, curve fitting and gray barycenter are used to extract the central pixel coordinates of the optical stripe. These methods have a simple calculation process, but they are easily affected by noise and have low accuracy. The gray-scale barycenter method [3,4,5,6,7,8,9,10,11,12] is simple in calculation and has been improved by researchers through combining it with other methods, such as the extremum method, principal component analysis, curve fitting algorithm, adaptive operation, mean value calculation, filtering, contour segmentation, etc. The gray-scale center of the gravity method combined with the image adaptive operation can also greatly reduce the processing time of images. Wang Fubin et al. [13] used image mask manipulation, an adaptive convolution template, the quadratic-weighted gray-scale center of gravity method and principal component analysis to obtain fringed subpixel coordinates. LU Yonghua et al. [14] segmented the strip area and obtained the width of the laser stripe by boundary detection. Then, they used the quadratic-weighted gray centroid and slope threshold optimization methods to complete the center extraction of the optical stripe. The improved method can achieve high-precision extraction of the optical stripe center at subpixel level. In the research of scholars, it is common to adjust the gray level by stripe pretreatment, to eliminate the noise by mean filtering and to extract the stripe center by curve fitting [15,16,17]. As a new research direction, deep learning [18,19] has been widely applied in the field of image processing and has been studied by many scholars in the applied scene of light stripe center extraction. Because a deep neural network with good feature learning and good anti-noise immunity can extract the whole distribution and bending features of laser stripes, it can segment the stripes very well. The BP neural network [20,21,22] is a part of deep learning. It can not only extract the center of the light stripe accurately and quickly but also effectively eliminate the influence of noise and reflection on the surface of the measured object.

There are other ways to make improvements. For example, Zhou Yuan et al. [23] used the density clustering algorithm to effectively narrow the search area and introduced the improved image seam algorithm based on the core points. Although the algorithm has a strong anti-interference ability, reliable precision and fast operation speed, it cannot achieve subpixel detection accuracy. In order to obtain the width of the light stripe region and to segment the light stripe, Southern [24] and other researchers used the threshold method to avoid the calculation of the non-light stripe region, and then detected the outline of the laser stripe by stochastic Hough linear transformation. In addition, the Steger algorithm was used to extract the center and splices of the stripe. Pan Shuo et al. [25] extracted the center of a light stripe by the skeleton thinning algorithm, then matched and grouped the image blocks to obtain the center coordinates of the light stripe in the image blocks. They considered the mean value as the center of the final light stripe. This method has good anti-noise performance, but it has not been tested in running time. Li Weiming [26,27] and others used threshold contour tracking to avoid the scanning of redundant regions, which greatly improves the running speed of the algorithm. However, this algorithm is not suitable for the situation of poor light quality. Then, a new algorithm based on mean shift is proposed, which has the advantages of high speed, good robustness and high precision. Liu Zhen et al. [28] used a gradient threshold to segment the effective region, took the maxima obtained from the cross-correlation operation as the initial center and extracted the center of the light stripe accurately through curve fitting. The algorithm has good robustness and anti-noise performance, but the precision is slightly lower when the gray-scale slowly changes place. Liu Changwen et al. [29] put forward a new method to modify the center of curve light. The new image, which is essentially similar to the linear light stripe, is composed of the center of the light stripe and the gray value of the pixels around it. Then, the zero-crossing detection of the first derivative of the new image is carried out to obtain the center point after correction. It can effectively correct the skew of curve light center caused by Gauss smoothing. Zhang Xiuhua et al. [30] extracted the center of a structured light stripe with a wide field of view by calculating the peak intensity value of each pixel and segmenting the image according to the threshold value related to the peak intensity. The center of a line-structured light stripe can also be extracted accurately under a complex background, uneven illumination and the field of view.

Although many algorithms have been proposed for line-structured light center extraction, these algorithms do not take into account the characteristics of light stripe distribution and the actual measurement accuracy of the workpiece. Therefore, these algorithms are unsuitable for different light stripe distribution images, and the measuring speed is slow. In this paper, an adaptive bidirectional gray-scale center of gravity extraction algorithm for laser stripes is proposed by analyzing the features of light stripes in different images. First, the initial image processing area is set according to the floating field of the light stripe in the camera. Then, the adaptive image processing area is set according to the actual position of the light stripe. Secondly, the center of light stripe is obtained by combining the upper contour with the barycenter of the bidirectional gray-scale. The center of light stripe is optimized by judging the slope to solve the deviation problem between adjacent center points. Finally, the slope and intercept are used to complete the breakpoint. To verify the running speed, measurement accuracy and adaptability of the proposed algorithm, comparative experiments are carried out between the proposed algorithm in this paper and Steger, the gray-scale center of gravity, the improved gray-scale center of gravity and geometric barycenter algorithms, which focus on four aspects, including the extraction effect, RMSD, standard deviation and average extraction time. In addition, the algorithm is used to process the image of the light stripes in different environments to verify the adaptability of the algorithm. Through these experiments, the algorithm is analyzed in detail, and the algorithm’s validity in extraction accuracy and running speed is verified.

## 2. An Adaptive Bidirectional Gray-Scale Center of Gravity Extraction Algorithm

**Figure 1:**
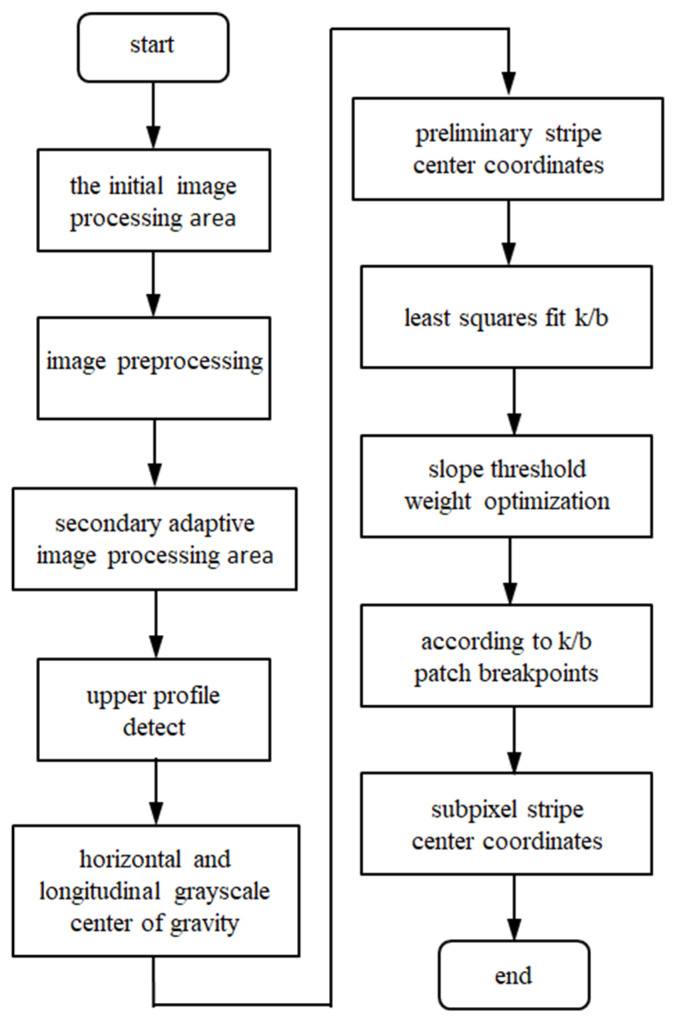
Algorithm flow chart.

In this paper, we propose an algorithm to extract the center of a light stripe, which sets the initial image processing area according to the floating field of the light stripe in the camera. Then, the adaptive image processing area is adopted to reduce the redundant scanning of the image area without the light stripe. Secondly, the initial center coordinates of the light stripe are obtained by combining the center of gravity of transverse gray-scale with the center of gravity of longitudinal gray-scale in three steps. The slope threshold is used to optimize the weight to solve the problem of the deviation between adjacent centers. Finally, the center line is completed by slope and intercept. The detailed flow of the algorithm is shown in Figure 1.

### 2.1. Light Distribution Characteristics

**Figure 2:**
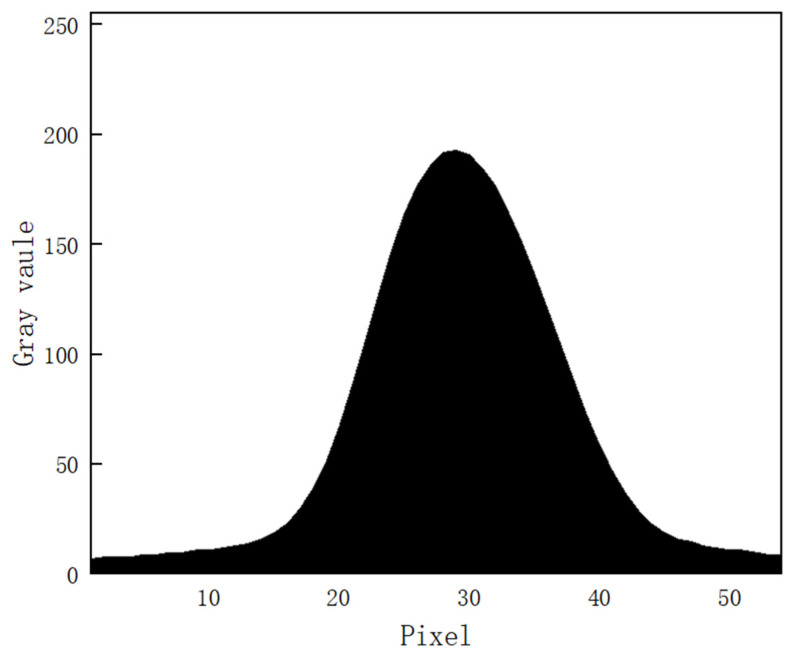
Gaussian distribution of light stripe cross-sections.

**Figure 3:**
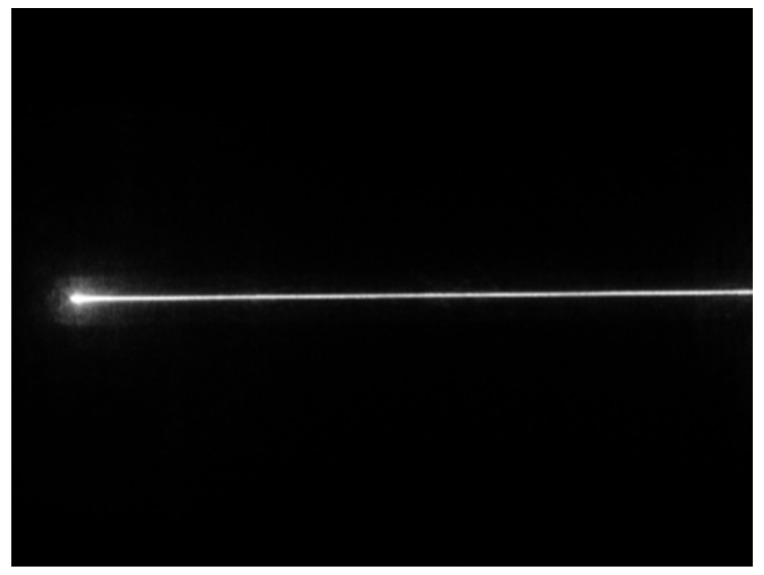
Light stripes.

**Figure 4:**
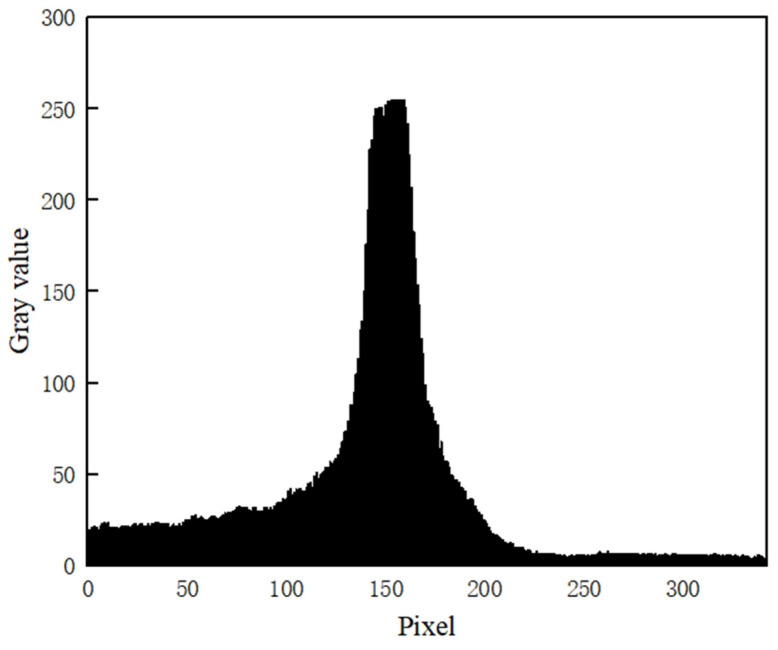
Cross section of light stripe with Gaussian-like distribution.

Under the conditions that ambient light is good, the laser light quality is uniform, and the surface material of the measured object is appropriate; the cross-section intensity of the light stripe emitted by the laser is Gaussian distribution, as shown in Figure 2. However, the quality of the actual light stripe is easily affected, and the detection environment in the factory is complex, resulting in problems such as a large slope of the light stripe, broken line, fold line, curve, etc. The photo of the light streaks taken under actual conditions is shown in Figure 3. Additionally, the distribution of pixel gray values of the cross-section of the light bar is shown in Figure 4. The contrast between Figure 2 and Figure 4 shows that the pixel gray values in the cross-section of the light stripes are not symmetrical and uniformly distributed, and the peak width of the gray values is not only one pixel.

### 2.2. Initial Image Processing Area Setting

In industrial detection, the line laser structure is usually used to detect a workpiece. The region of the light stripe is basically fixed, so the image can be set in the region. However, there is mechanical vibration in a real factory detection environment, which causes the light stripe to float in the image. Therefore, the primary image processing area is set in this paper, which not only improves the running speed of image processing but also adapts to the floating range of the field of the light stripe.

**Figure 5:**
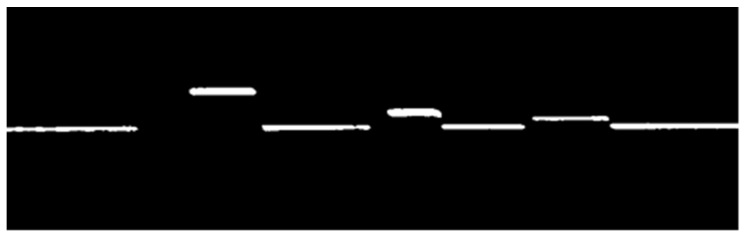
Light stripe area on the surface of the calibration blocks.

As shown in Figure 5, three kinds of calibration blocks (3, 5, 10 mm) were used in the experiment. In industrial detection, the thickness of the workpiece is not uniform, being accompanied by the mechanical vibration, which will cause the light stripe to float in the image. Therefore, before image pre-processing, the initial image processing region is set according to the size range of the measured object, which can improve the speed of image processing without filtering out the useful information of light stripes.

### 2.3. Image Preprocessing

In order to remove some useless interference information in the image, improve the detectability of useful information, find useful information and simplify these useful data, pre-processing operations such as gray-scale processing, binarization and Gauss filtering are used to denoise and enhance the optical stripe image in this paper.

Gray-scale processing is a pre-processing process that transforms the image from color to gray level. According to Formula (1), the R, G and B values of the color image are calculated to obtain the gray-level image, which has achieved the processing effect of image enhancement.

(1) Gray=0.3R+0.59G+0.11B

In the formula, Gray represents the gray value, and R, G and B represent the red, green and blue channel values of the color image, respectively.

In order to reduce the image noise and eliminate some useless interference noise in the image, the Gauss filtering method, which is a linear smoothing filter to reduce the image noise, is adopted, as shown in Formula (2).

(2) $$f(x)=\frac{1}{\sqrt{2\pi }\sigma }\exp(-\frac{{\left(x-\mu \right)}^{2}}{2\sigma^{2}})$$

In the formula, f(x) represents the probability, σ represents the standard deviation, μ represents the coordinate value of the curve peak center and $\frac{1}{\sqrt{2\pi }\sigma }$ represents the height of the curve peak.

In order to reduce the amount of data to be processed and find the upper contour of the light stripes conveniently, it is necessary to binary the gray image. The binarization process is the change in an image from gray or color to black and white, corresponding to two gray values, i.e., 0 and 255. The processing formula is shown in Formula (3).

(3) $$Gray(u,v)=\left\{ \begin{array}{l} 1,g(u,v)\;>\;t\\ 0,g(u,v)\leq t \end{array}\right.$$

In the formula, Gray(u,v) denotes the Gray value corresponding to the binarization of pixels (u,v), g(u,v) denotes the Gray value corresponding to pixels (u,v) and t denotes the global threshold.

### 2.4. Secondary Adaptive Image Processing Region Setting

After setting the initial image processing region, the second adaptive region is set to locate the specific position coordinates of the light stripe in the image, which can further reduce the redundant scanning of the image region without the light stripe and speed up the running rate.

**Figure 6:**
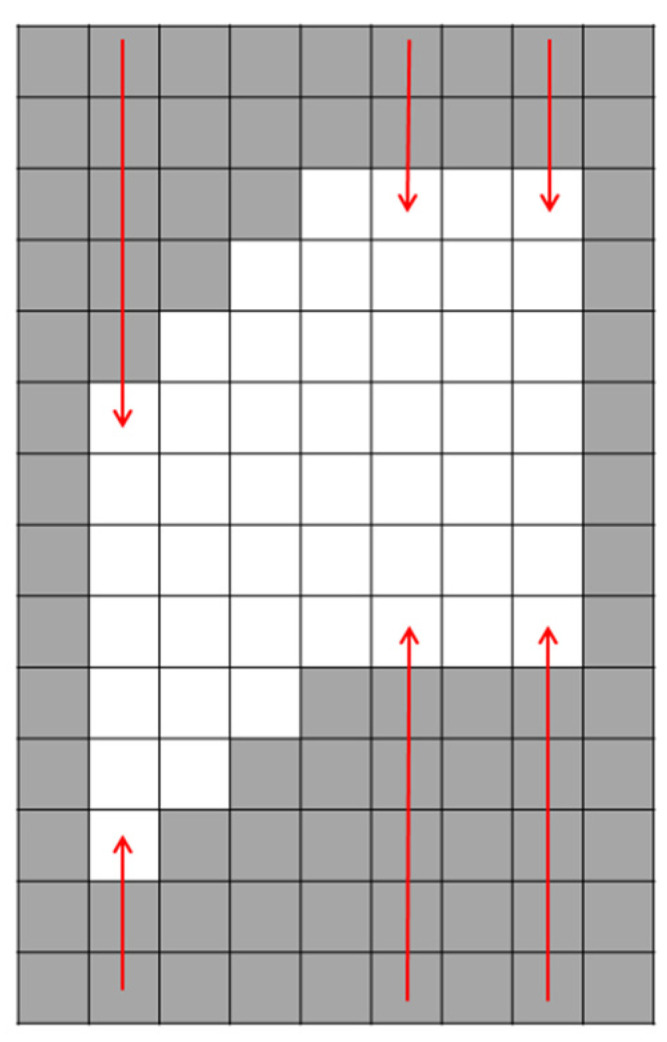
Schematic of the secondary adaptive image processing area.

**Figure 7:**
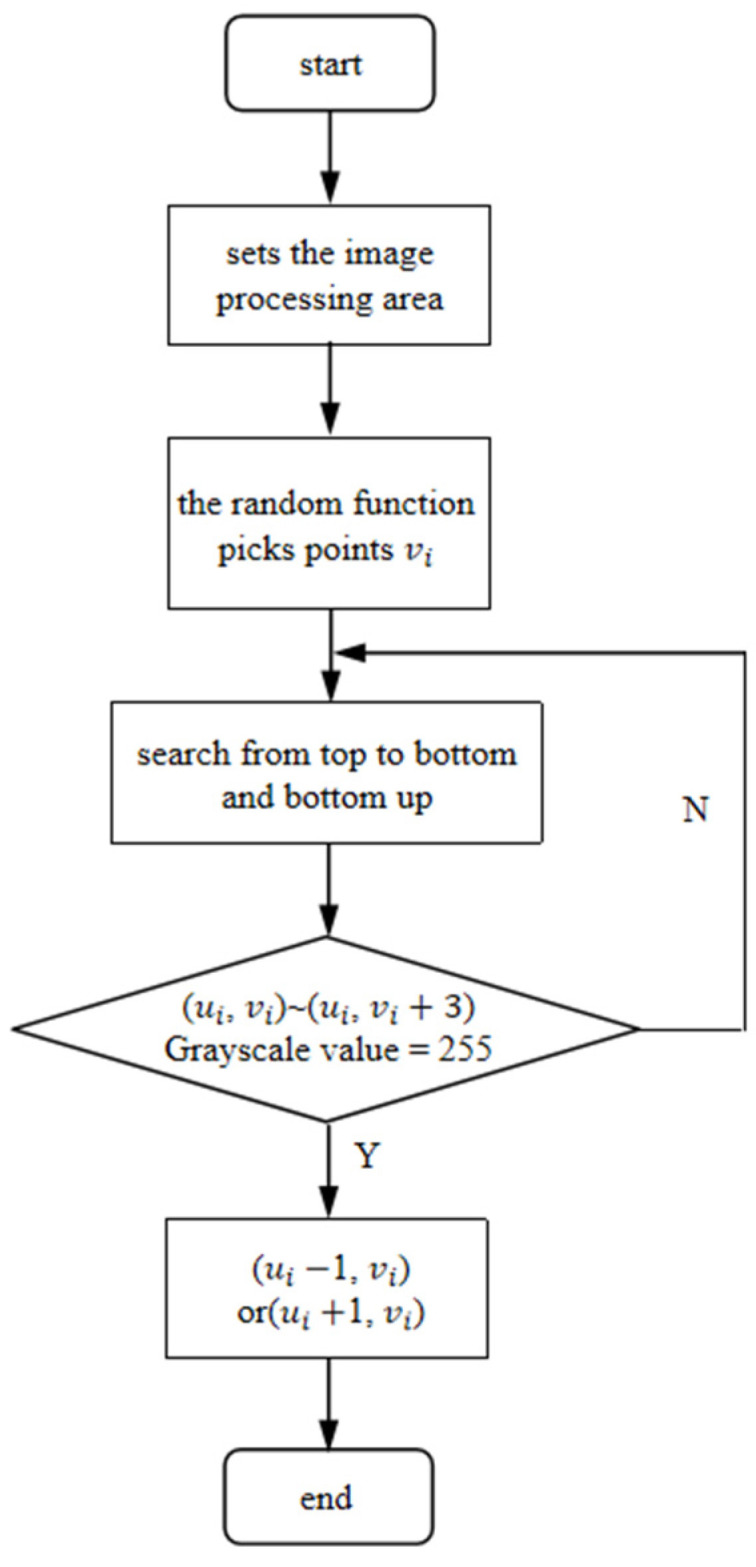
Flow chart of the secondary adaptive image processing area.

The second adaptive image processing region is set on the basis of the initial image processing region. Additionally, the algorithm selects the random points $v_{i}$ by using the random function based on the binary image. The number of random points increases with the increase in image resolution. Then, there is a top-down and bottom-up search for edge points that meet the threshold condition. The threshold condition of the edge point is that the gray-level threshold of the point and three consecutive points in the column direction are 255. Then, the top and bottom points of the edge are selected to extend a point. The range of the extension points is set as the range of the secondary image processing area. A schematic diagram of the secondary adaptive image processing area setting is shown in Figure 6, and a flowchart is shown in Figure 7.

### 2.5. Principle of Bidirectional Center of Gravity Method

The barycenter method is to scan by column or row. The centroid of the gray value of the column or row is calculated by the gray-weighted algorithm, and its centroid is taken as the center of the light stripe. The pixel coordinate satisfying the threshold condition is $u_{i}/v_{i}.$ $f_{i}$ is the gray value corresponding to the coordinate point in the image, and the centroid position of the column or row is obtained as $\overset{-}{u}/\overset{-}{v}$. The formula is as following:

(4) $$\left\{ \begin{array}{l} \bar{u}=\frac{\sum_{i=0}^{m}u_{i}\times f_{i}}{\sum_{i=0}^{m}f_{i}}\\ \bar{v}=\frac{\sum_{i=0}^{m}v_{i}\times f_{i}}{\sum_{i=0}^{m}f_{i}} \end{array}\right.$$

**Figure 8:**
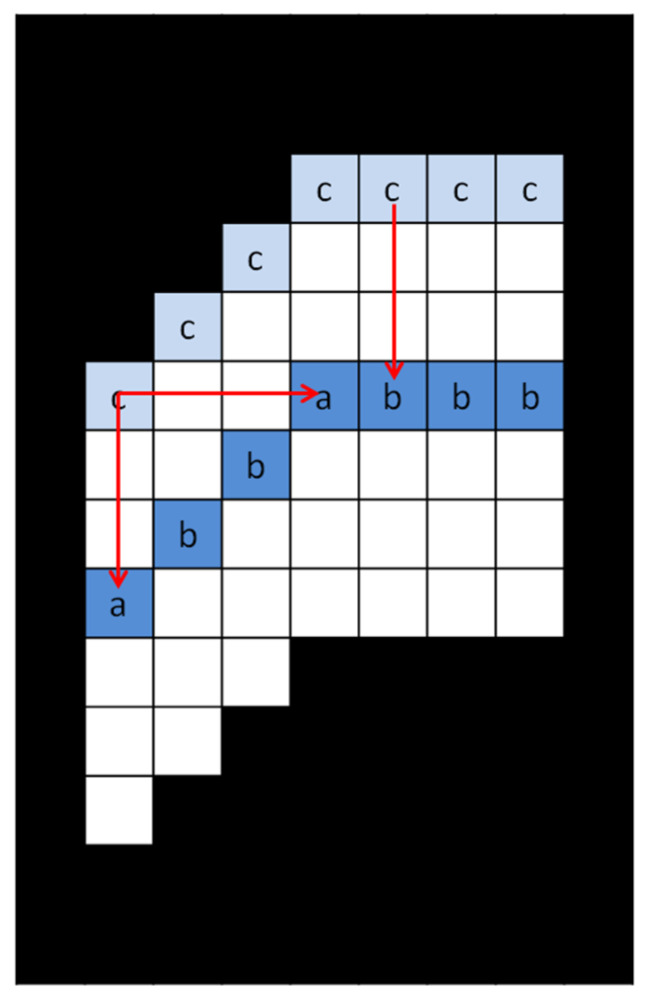
Principle diagram of bidirectional center of gravity method.

**Figure 9:**
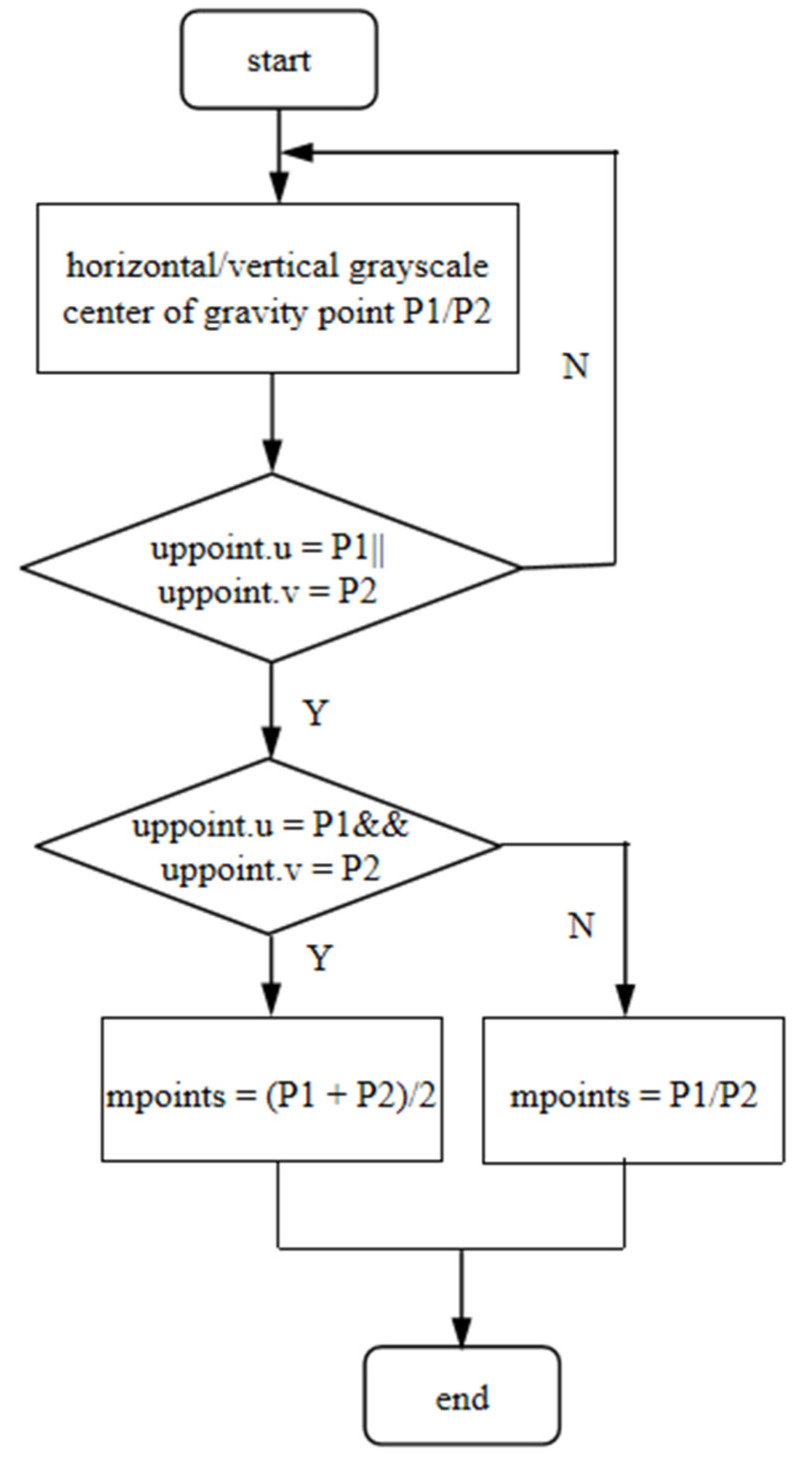
Flow chart of bidirectional center of gravity method.

The gray-scale center of gravity method is used to calculate the horizontal and vertical centers of light stripes. However, in the case of a large tilt angle, the single-direction gray-scale center of gravity method will result in the loss of some center points of light stripes; it is not suitable for the center extraction of all optical stripes. On the basis of the gray-scale center of gravity method, a bidirectional center of gravity algorithm is proposed in this paper. A schematic diagram is shown in Figure 8. Each upper contour point corresponds to a light stripe center point. If a center point exists in only one direction, the center point is retained. If there are two central points at the same time, the method of taking the average value preserves a central point coordinate. In Figure 8, c is the uppoint of the upper contour points of the light stripe, a is the existence of both horizontal and vertical search points and b is the existence of only one point in the horizontal and vertical search points. The flowchart is shown in Figure 9.

The bidirectional center of gravity flow chart is as follows.

(1) First, the horizontal and vertical gradation barycenter of the image are recorded respectively, and the set of the barycenter is called P1/P2.
(2) Then, the horizontal and vertical search is carried out according to the uppoint. The searching distance is defined as d1/d2, respectively, and the searching range does not exceed the width τ of the half-light stripe. The center points in P1/P2 that meet the search conditions are retained, and the ones that do not meet the search conditions are eliminated.
  (5) $$\begin{array}{l} \left\{0\leq d_{1}\leq \frac{\tau }{2},0\leq d_{2}\leq \frac{\tau }{2}\right\}\\ \begin{array}{c} d1=\left|uppoint-P1\right|\\ d2=\left|uppoint-P2\right| \end{array} \end{array}$$
(3) If both horizontal and vertical search points exist, the middle point of the coordinates of two points P1/P2 will be counted as the mpoints of the middle point of the light stripe, as shown in Formula (6). Otherwise, the point of the horizontal or vertical search will be counted as the mpoints.
  (6) $$mpoints=\left\{\begin{array}{l} (P1(u,v)+P2(u,v))/2\\ P1(u,v)/P2(u,v) \end{array}\right\}$$

In Formula (6), P1(u,v) is the image coordinate of P1 and P2(u,v) is the image coordinate of P2.

### 2.6. The Principle of Weight Optimization

The center coordinates of the light stripe extracted by the gray-scale barycenter method have inflection points between adjacent center points, which are not smooth and have a large deviation. If the offset is large, it will affect the precision of the center coordinate of the light stripe. In order to solve this problem, the weight optimization algorithm is used to adjust the center coordinates.

Using the least squares method to fit the coordinates of the center point obtained by the improved barycenter method, the slope k and intercept b are obtained. k is used as the judging threshold. Then, the slope $k_{1}$ between the adjacent centers $y_{i}$ and $y_{i+1}$ is calculated sequentially, and when $k_{1}$ exceeds the slope threshold k, the $y_{i+1}$ is optimized by weighting.

(7) $$\begin{array}{l} Xsum=sum(x_{1}+x_{2}+\ldots +x_{n})/n\\ Ysum=sum(y_{1}+y_{2}+\ldots +y_{n})/n\\ Xx=sum((x_{i}-Xsum{)}^{2})\\ Xxy=sum((x_{i}-Xsum)*(y_{i}-Ysum))\\ k=Xxy/Xx\\ b=Ysum-k*Xsum \end{array}$$

yi+1=yi+1                    ,k1≤ka1∗yi−1+a2∗yi+a3∗yi+2+a4∗yi+3        ,k1>k

In Formula (7), a1,a2,a3,a4 are the weight coefficients.

**Figure 10:**
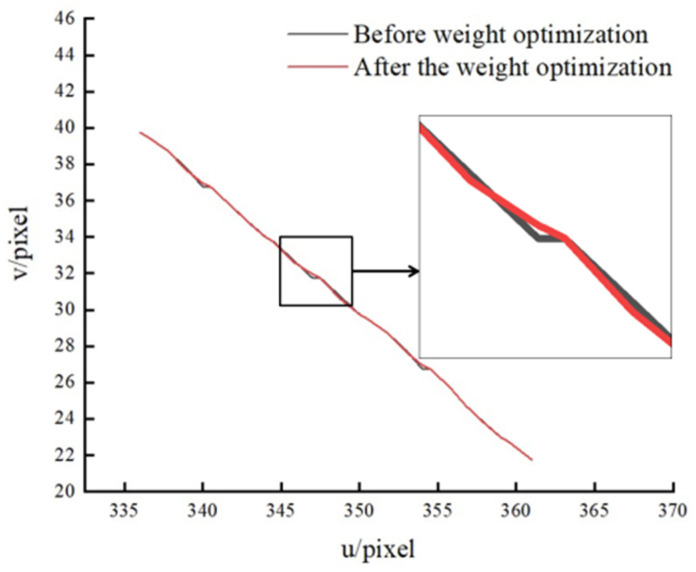
Weight optimization comparison chart.

The contrast figure of the light stripe center point distribution before and after weight optimization is shown in Figure 10. From the change in the fold line between the center points, it can be seen that the line segment of the center point is much smoother than before optimization. This is helpful to improve the accuracy of the center point.

## 3. Experimental Results and Analysis

Contrast experiments were carried out between the proposed algorithm and other traditional algorithms, including Steger, the gray center of gravity, geometric center and the improved gray-scale center of gravity algorithm. The Steger algorithm uses Hessian matrix and Taylor expansion to area the center of a light stripe. For the gray center of gravity algorithm, the centroid of gray values distribution in the direction of columns or rows is recorded as the center point of the light stripe. The geometric center algorithm takes the center of the contour points on both sides as the center point of the light stripe. The improved gray-scale center of gravity algorithm adopts a subpixel adaptive extraction algorithm for linear structure light stripe center. On the basis of the mask operation, the optical stripe center is obtained by the quadratic-weighted gray-scale center of gravity, regional growth and principal component analysis.

**Figure 11:**
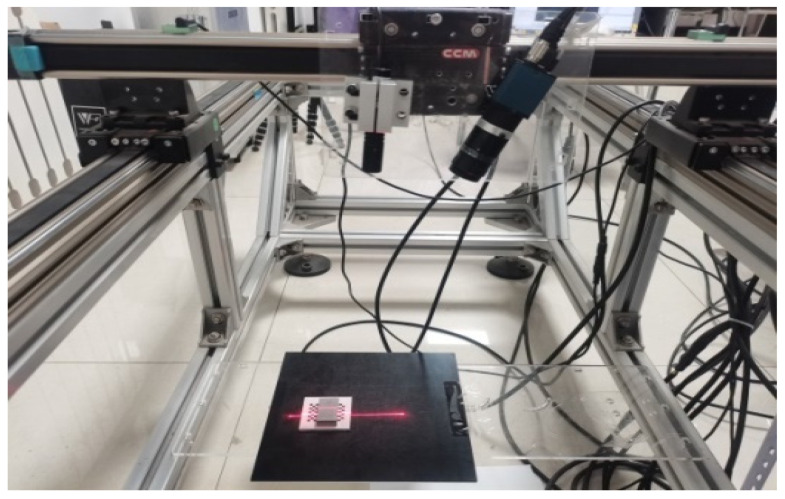
Experimental platform diagram.

**Figure 12:**
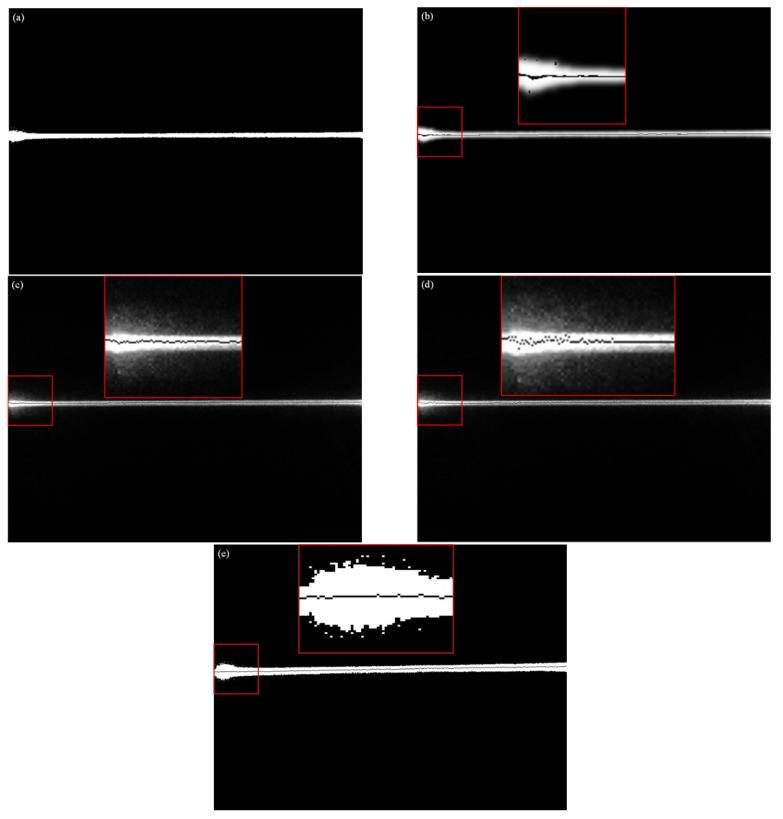
The original image of the light stripe, and the center graph of the light stripe extracted by each algorithm. (**a**) The original image of the light stripe; (**b**) Steger; (**c**) the gray-scale center of gravity; (**d**) geometric center; and (**e**) the improved gray-scale center of gravity.

**Figure 13:**
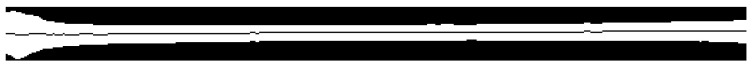
The center graph of the light stripe extracted by the proposed algorithm.

Figure 11 shows the experimental platform we built. For the experiment, the CPU used was Intel (R) Core (TM) i7-9700F CPU@3.00 GHz with 16 GB of memory. In this experiment, we selected VS2015 as the development platform and called OpenCV to complete the optimization of the algorithm. The camera used in the experiment was a MER-051-120GM/C-P, and the image resolution was 808 × 608 pixel. The experimental results obtained by the presented algorithm in this paper were compared with that of the traditional gray-scale barycenter method, the geometric center method, the improved gray-scale center of gravity algorithm and the Steger algorithm. The display results in the image were rounded to subpixels. Figure 12 shows the original image of the light stripe and the central image of the light stripe (including the local magnification image) extracted by the algorithms. Figure 13 is the light stripe center graph extracted by the new algorithm.

As shown in Figure 12 and Figure 13, there are some outer points in the center of the light stripe extracted by the Steger algorithm, which affects the precision. The center point of the light stripe extracted by the gray-scale center of gravity algorithm fluctuates greatly, and the accuracy will become very low when the center slope of the extracted light stripe is large. The center of the light stripe extracted by the geometric center algorithm has a certain deviation. Especially, when the image has a lot of noise, the deviation will be very large. The center of the light stripe extracted by the improved gray-scale center of the gravity algorithm has a slight fluctuation, and the extraction effect is also very good. There is no outer point in the center of the light stripe extracted by this algorithm, and all the center points are extracted with good continuity and smoothness.

The accuracy of the algorithm was further analyzed, and the difference between the measured values is calculated by the RMSD. The RMSD represents the difference between the mean of the center point and the center point of each light stripe. The root-mean-square deviation (RMSD) of each algorithm is shown in Table 1. The RMSD is

(8) $$RMSD=\sqrt[{}]{\frac{\sum_{i=1}^{n}{\left(y_{i}-y\right)}^{2}}{n}}$$

where n is the number of center points, $y_{i}$ is the predicted value of the first center point and y is the true center of each center point.

It can be seen from Table 1 that the RMSD of the proposed algorithm is the smallest, and the accuracy of the center point of the light stripes extracted by the proposed algorithm is the best.

The standard deviation can be used as a measure of uncertainty and is suitable for reflecting the discreteness of data. First, the center pixel coordinates of the light stripe extracted by the algorithm are fitted to a straight line by the least square method. The formula is as following:

(9) $$Ax_{i}+By_{i}+C=0$$

where A, B and C are the coefficients obtained by fitting the linear equation. $\left(x_{i},y_{i}\right)$, corresponding to the image coordinates of each central point, calculate the distance $d_{i}$ from the center pixel coordinates on the line, which is

(10) $$d_{i}=\left|\frac{Ax_{i}+By_{i}+C}{\sqrt{A^{2}+B^{2}}}\right|$$

The standard deviation of the distance from the center pixel coordinates to the line extracted by the algorithm represents the accuracy of the center pixel coordinates extracted by the algorithm. n is the number of center points, d is the average value of the distance from all the center pixel coordinates to the line and the standard deviation ε of the center pixel coordinates to the line is

(11) $$\varepsilon =\sqrt{\frac{\sum_{i=1}^{n}{\left(d_{i}-d\right)}^{2}}{n}}$$

The standard deviation of each algorithm is shown in Table 2. Compared with the other four algorithms, the standard deviation of the proposed algorithm is the smallest, and the precision of the center pixel coordinates is the best.

**Figure 14:**
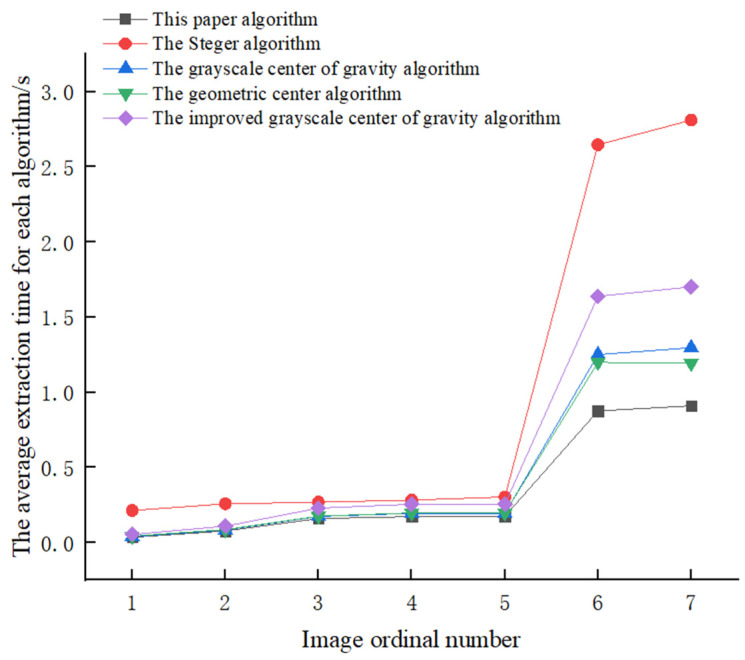
Dashed plot of the algorithms’ average extraction time.

In order to verify the extraction speed of this algorithm, we tested the images with different laser line width by using each algorithm, and the images with different resolution were collected in seven different conditions. No less than 20 images were taken in each case, and the average extraction time of each case is calculated. The average extraction time of each algorithm is shown in Table 3. The average extraction time of each algorithm was transformed into the polyline in Figure 14, which makes the observation more intuitive.

The experimental data show that the speed of this algorithm is 2.82 times faster than that of the Steger algorithm, 1.34 times faster than that of the gray centroid algorithm, 1.29 times faster than that of the geometric centroid algorithm and 1.76 times faster than that of the improved gray-scale center of gravity algorithm. It can be seen from the polyline in Figure 14 that the time to extract the center of the laser stripe by using this algorithm increases with the increase in the image resolution. Compared with other algorithms, the speed-up effect is not significant when the image resolution is small, but the speed-up effect will become more significant with the increase in image resolution.

**Figure 15:**
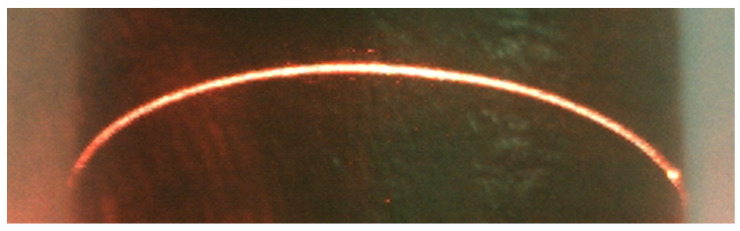
Curve laser stripe.

The laser stripes are all straight lines. In order to verify the accuracy of the algorithm, a group of curve laser stripes were analyzed by the RMSD. The laser stripes of the curve are shown in Figure 15, and the RMSD calculated by each algorithm is shown in Table 4.

Compared with the experimental data in the table above, the proposed algorithm has high accuracy and is suitable not only for the center extraction of linear laser stripe but also for the center extraction of curvilinear laser stripe.

**Figure 16:**
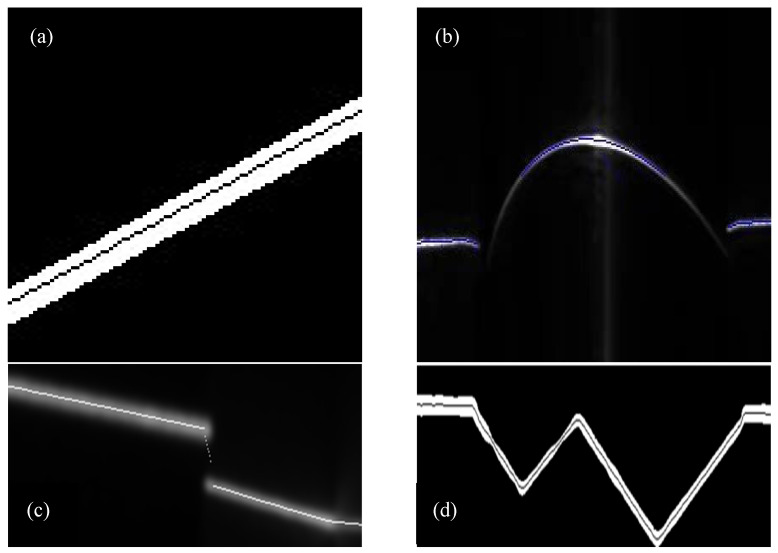
The algorithm in this paper processes the renderings. (**a**) Steel plate surface; (**b**) upper surface of roller; (**c**) paperboard surface; and (**d**) angle steel upper surface.

In order to verify the applicability and anti-noise of the algorithm, some images were collected in the complex detection environment of the factory. The images were processed by the proposed algorithm. Figure 16 is the result of the algorithm.

It can be seen from Figure 16 that the algorithm presented in this paper has effective results, wide applicability and a certain degree of anti-noise.

## 4. Conclusions

In this paper, an adaptive bidirectional gray-scale center of gravity extraction algorithm for laser stripes was proposed, and the core of the algorithm was described in detail. The algorithm preserves only the light stripe region of the image as far as possible. At the same time, the center point of the light stripe is also preserved by using the method of contour and gray barycenter so that the speed, precision and adaptability of the center extraction of the light stripe are improved. The experimental results show that the proposed algorithm is faster and more accurate than Steger, gray-scale barycenter, the improved gray-scale center of gravity and geometric centroid algorithms. The algorithm is suitable for the cases of the large slopes, broken lines, fold lines and curves of light stripes. The algorithm’s speed is 2.82 times faster than that of Steger algorithm and 1.34 times faster than that of gray centroid method. The standard deviation of extraction accuracy reached 0.314051 pixels. The algorithm in this paper has high measurement accuracy and adaptability in practical detection.

## Figures and Tables

**Table 1:** The RMSD difference of each algorithm.

This Paper’s Algorithm	Steger	Gray-Scale Center of Gravity	Geometric Center	Improved Gray-Scale Center of Gravity
0.3813	1.9510	0.5678	0.6000	0.4752

**Table 2:** The standard deviation of each algorithm.

This Paper’s Algorithm	Steger	Gray-Scale Center of Gravity	Geometric Center	Improved Gray-Scale Center of Gravity
0.314051	1.824630	0.391013	0.428601	0.361370

**Table 3:** The average extraction time for each algorithm.

Image Ordinal Number	Image Resolution/Pixel	Processing Time/s				
		This Paper’s Algorithm	Steger	Gray-Scale Center of Gravity	Geometric Center	Improved Gray-Scale Center of Gravity
1	317 × 214	0.034	0.213	0.038	0.042	0.053
2	649 × 343	0.077	0.257	0.083	0.086	0.109
3	808 × 550	0.159	0.268	0.173	0.175	0.228
4	808 × 600	0.172	0.282	0.194	0.197	0.254
5	808 × 608	0.175	0.303	0.194	0.197	0.255
6	4096 × 2500	0.875	2.647	1.250	1.199	1.639
7	4096 × 3000	0.910	2.811	1.296	1.192	1.701

**Table 4:** The RMSD difference of each algorithm for curve.

This Paper’s Algorithm	Steger	Gray-Scale Center of Gravity	Geometric Center	Improved Gray-Scale Center of Gravity
1.5833	1.6690	1.9980	2.1131	1.8017

## Data Availability

Not applicable.
